# Anti-reflux medication use in preterm infants

**DOI:** 10.1038/s41390-021-01821-y

**Published:** 2021-10-29

**Authors:** Haslina Binti Abdul Hamid, Lisa Szatkowski, Helen Budge, Shalini Ojha

**Affiliations:** 1 Population and Lifespan Sciences, School of Medicine, University of Nottingham, Nottingham, UK; 2 Dietetic Programme, Faculty of Health Sciences, Universiti Kebangsaan Malaysia, 50300 Kuala Lumpur, Malaysia; 3 Neonatal Unit, University Hospitals of Derby and Burton, Derby, DE23 3DT UK

## Abstract

**Background:**

Current recommendations do not support the use of anti-reflux medications to treat gastro-oesophageal reflux disease (GORD) among preterm infants.

**Objective:**

To describe the prevalence of GORD and the use of anti-reflux medications amongst very preterm infants (<32 weeks’ gestational age (GA)) in neonatal units in England and Wales.

**Design:**

Retrospective cohort study using the National Neonatal Research Database.

**Results:**

Among 58,108 infants [median GA (IQR) 29 (27–30) weeks], 15.8% (*n* = 9191) had a diagnosis of GORD and 36.9% (*n* = 12,446) received anti-reflux medications. Those who received anti-reflux medications were more preterm [GA, median (IQR): medications, 28 (26–30) vs. no medications, 30 (28–31); *p* < 0.001] and had lower birth weight [mean (SD): medications, 1124 g (354) vs. no medications, 1265 g (384); *p* < 0.001]. Most (57%, *n* = 12,224) received Gaviscon, or Histamine-2 Receptor Antagonist (H2RA) (56%, *n* = 11,959). Over time, prokinetic use has declined substantially, the use of H2RAs and Gaviscon has reduced although they continue to be used frequently, whilst the use of PPIs has increased.

**Conclusions:**

Anti-reflux medications are frequently prescribed in very preterm infants, despite evidence to suggest that they are not effective and may be harmful. Clear guidelines for diagnosing GORD and the use of anti-reflux medications are required to rationalise the pharmacological management of GORD in preterm infants.

**Impact:**

- Anti-reflux medications are frequently prescribed, often without a diagnosis of gastro-oesophageal reflux disease, to very preterm infants while in the neonatal unit and at discharge.
- Half of the infants born at <28 weeks’ gestational age receive anti-reflux medications in hospital and a quarter are discharged home on them.
- Although the use of prokinetics declined following alerts of adverse events, histamine2-receptor antagonists and alginates such as Gaviscon continue to be used and the use of proton-pump inhibitors has increased more than 2-fold.

## Introduction

Gastro-oesophageal reflux (GOR), the passage of gastric contents into the oesophagus, is common in preterm infants.^1^ It usually occurs due to the relatively abundant volume of liquid intake and supine feeding position. However, GOR can worsen and lead to troublesome signs and symptoms i.e., gastro-oesophageal reflux disease (GORD).

Determination of the exact prevalence of GOR versus GORD is challenging because of an unclear distinction between physiologic and pathologic reflux. Additionally, the terms “reflux”, “acid-reflux”, and GORD are often used interchangeably by healthcare professionals as well parents and families of infants.^2^ In a retrospective cohort study of 33 neonatal units in the USA,^3^ there was a wide variation between the units in the proportion of preterm infants who received a diagnosis of GORD based on the International Classification of Diseases (ICD-9). Approximately 10% of infants born at 22−36 weeks gestational age (GA) were recorded to have GORD with diagnosis prevalence ranging from 2.4 to 29.9% across the included neonatal units.

Anti-reflux medications, including Histamine-2 receptor antagonists (H2RA) and proton-pump inhibitors (PPI), are unlicensed for use in neonates in the UK and many other countries,^4^ though *off-label* use is frequently reported.^5^ Antacids and other acid-suppressing agents may reduce gastric acidity but anti-reflux medications do not improve signs/symptoms of GORD and their use has been associated with increased risks of adverse outcomes including necrotising enterocolitis (NEC) and infections.^6^ The North American Society for Pediatric Gastroenterology, Hepatology, and Nutrition and European Society for Paediatric Gastroenterology Hepatology and Nutrition (NASPGHAN and ESPGHAN combined) clinical practice guidelines^1^ suggests non-pharmacological approaches to GORD before pharmacological therapy. Similarly, the American Academy of Paediatrics recommends that anti-reflux medications should be used with caution, if at all, in preterm infants due to lack of evidence of efficacy and possible significant harm.^7^

Despite these recommendations, anti-reflux medications are frequently used. A large retrospective study including infants admitted to neonatal units in 43 children’s hospitals in the USA reported that 24% received either an H2RA or PPI with the extremely preterm infants being the most likely to receive these medicines.^8^ Similarly, Malcolm et al.^9^ reported that a quarter of extremely low birth weight infants who were enrolled in the National Institute of Child Health and Human Development Neonatal Network generic database in 2002–2003 were discharged from the hospital with anti-reflux medications.

In the UK, two survey studies of neonatal healthcare professionals, in 2004^10^ and 2018,^11^ revealed that clinicians reported frequently prescribing anti-reflux medications in neonatal units. Dhillon and Ewer^10^ reported that, in 2004, nearly all respondents used anti-reflux medications to manage GOR. In 2018, another survey showed that the use of anti-reflux medications remained popular^11^ despite the increasing evidence of lack of efficacy and possible harm. Both studies analysed the use of medications as reported by clinicians. There are no studies that have analysed the prevalence of GORD diagnosis and the actual use of anti-reflux medications in neonatal units in the UK.

In this study, we aimed to describe the prevalence of GORD diagnosis and use of anti-reflux medications amongst very preterm infants in England and Wales. We also aimed to analyse the types of medications used, duration and trend of medications use between 2010 and 2017.

## Methods

We conducted a retrospective observational cohort study using the data held in the National Neonatal Research Database (NNRD). The NNRD is a repository of clinical data of admissions to the 200 neonatal units in England, Wales and Scotland that make up the UK Neonatal Collaborative.^12^

### Identification of study cohort

Infants born <32 weeks’ gestational age (GA), in England and Wales from 2010 until 2017, whose data are held in the NNRD were included. Infants were excluded if they were missing data on sex, birth weight, month of birth or one or more days of care, were a late admission to neonatal care (>24 h) or had an extreme birth weight for GA (*Z*-score ≥ +4 SD or ≤−4SD).

### Data extraction

Records of GORD were extracted by searching for specified terms in the “principal diagnosis at admission”, “daily diagnoses” and “diagnoses at discharge” fields. A list of anti-reflux medications for GORD was prepared according to the British National Formulary (BNF) and included Ranitidine (H2RA), Omeprazole and Lansoprazole (PPI), Domperidone, Metoclopramide, and Erythromycin (Prokinetics). Indicator variables were created for each day of care to show whether an infant was prescribed any of the anti-reflux medications. Code lists are available from the authors on request.

### Data analysis

Statistical analysis was performed using STATA 16.0 software (Stata Corp. College Station, TX). Descriptive statistics were used to describe infant characteristics, the prevalence of GORD and the use of anti-reflux medications. Values are presented as numbers and percentages for categorical data and for continuous variables, mean [±standard deviation (SD)] used for normally distributed data and median [interquartile range (IQR)] for non-normally distributed data. Linear regression was used where appropriate to quantify trends over time in medication use.

## Results

Among the total of 58,108 infants, 69.9% (*n* = 40,607) were born at 28–31 weeks GA and 30.1% (*n* = 17,501) were born at <28 weeks GA. 45.8% (*n* = 26,596) were female. Median (IQR) GA was 29 weeks (27–30), and the mean (SD) birth weight was 1213 g (379). The median (IQR) length of hospital stay was 49 days (34–74) and median (IQR) postmenstrual age (PMA) at discharge was 36 weeks (35–38).

Table 1 shows that 15.8% (*n* = 9191) of total infants had any record of GORD. Infants first received GORD diagnosis at median (IQR) PMA of 36 (33–38) weeks. Infants with a diagnosis of GORD were more preterm than infants without a diagnosis [GA, median (IQR): GORD, 28 weeks (26–30) vs. no GORD, 29 weeks (27–31); *p* < 0.001], had lower birth weight [mean (SD): GORD, 1114 g (337) vs. no GORD, 1232 g (384); *p* < 0.001] and fewer were female [*n* (%): GORD, 3856 (42.0%) vs. no GORD, 22,740 (46.5%); *p* < 0.001].

**Table 1:** Diagnosis of gastro-oesophageal reflux disease (GORD) and use of anti-reflux medications in infants born at <32 weeks’ gestational age in England and Wales from 2010 to 2017.

	All infants *n* = 58,108	<28 weeks’ gestation *n* = 17,501	28–31 weeks’ gestation *n* = 40,607
Any record of GORD diagnosis, *n* (%)	9191 (15.8)	3951 (22.6)	5240 (12.9)
PMA (weeks) at first GORD diagnosis, median (IQR)	36 (33–38)	36 (32–39)	35 (33–37)
Received anti-reflux medications in hospital, *n* (%)	21,446 (36.9)	8634 (49.3)	12,812 (31.6)
PMA (weeks) at first anti-reflux prescription, median (IQR)	32 (30–34)	30 (28–34)	32 (31–34)
Number of days of anti-reflux medications, median (IQR)	20 (9–37)	27 (11–49)	18 (9–30)
Receiving anti-reflux medications at discharge, *n* (%)	10,558 (18.2)	4352 (24.9)	6206 (15.3)

*GORD* gastro-oesophageal disease, *PMA* postmenstrual age, *IQR* interquartile range.

Of all the infants, 36.9% (*n* = 21,446) received anti-reflux medications during admission and 18.2% were receiving them at discharge (Table 1). However, among those who received anti-reflux medications, only 39.6% had a diagnosis of GORD. The PMA [median (IQR)] at first prescription of an anti-reflux medication was 32 weeks (30–34) and the treatments were continued for a median (IQR) duration of 20 days (9–37). 49.3% (*n* = 8634/17,501) of extremely preterm infants received anti-reflux medications during admission and 24.9% (*n* = 4352/17,501) were receiving them at discharge. Infants who received anti-reflux medications were more preterm [GA, median (IQR): medications, 28 (26–30) vs. no medications, 30 (28–31); *p* < 0.001], had lower birth weight [mean (SD): medications, 1124 g (354) vs. no medications, 1265 g (384); *p* < 0.001] and fewer were female [*n* (%): medications, 9172 (42.8%) vs. no medications, 17,424 (47.5%); *p* < 0.001].

Table 2 shows that among all infants who received anti-reflux medications (36.9%, *n* = 21,466), the largest number had Gaviscon (57%, *n* = 12,224) followed by H2RA (55.8%, *n* = 11,959), prokinetics (45.4%, *n* = 9734) and PPI (16.2%, *n* = 3480). The use of these medications among the subgroups of <28 weeks’ and 28–32 weeks’ GA is given in Table 2. Extremely preterm infants received all types of medications more frequently and for longer.

**Table 2:** Types of anti-reflux medication and number of days prescribed in infants born at <32 weeks’ gestational age in England and Wales from 2010 to 2017.

Received anti-reflux medications			
Types of medications and number of days prescribed	Total infants *n* = 21,466	<28 weeks’ gestation *n* = 8634	28–31 weeks’ gestation *n* = 12,812
Gaviscon, *n* (%)	12,224 (57.0)	4467 (51.7)	7757 (60.5)
Number of days, median (IQR)	17 (8–29)	21 (10–37)	15 (8–25)
PPI, *n* (%)	3480 (16.2)	1736 (20.1)	1744 (13.6)
Number of days, median (IQR)	20 (10–34)	24 (11–41)	18 (9–29)
H2RA, *n* (%)	11,959 (55.8)	5533 (64.1)	6426 (50.2)
Number of days, median (IQR)	15 (6–30)	17 (7–35)	14 (6–26)
Prokinetics, *n* (%)	9734 (45.4)	4231 (49.0)	5503 (43.0)
Number of days, median (IQR)	21 (10–37)	27 (12–48)	19 (9–31)

*PPI* proton-pump inhibitor, *H2RA* H2 receptor antagonists, *IQR* interquartile range.

During their stay, 11.4% (*n* = 6624) infants received two and 6.4% (*n* = 3687) received three types of anti-reflux medications. 16.7% (*n* = 9694) of infants received two or more anti-reflux medications on the same day of care and 8.6% (*n* = 4986) of infants were on two or more anti-reflux medications at discharge.

**Fig. 1:**
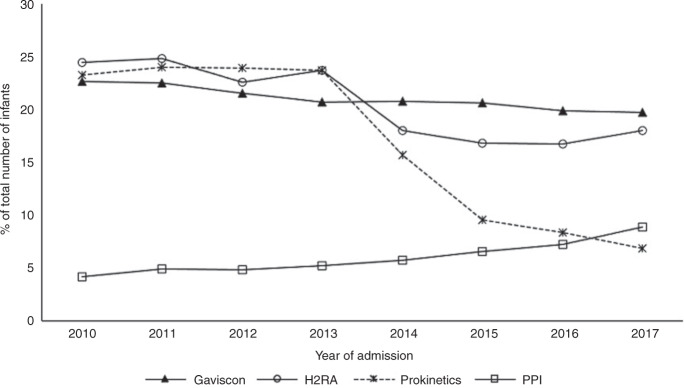
Trends in prescribing anti-reflux medications among infants born at <32 weeks’ gestational age in England and Wales from 2010 to 2017. H2RA histamine-2 receptor antagonists, PPI proton-pump inhibitors.

The change in prescribing of anti-reflux medications between 2010 and 2017 is shown in Fig. 1. The use of prokinetics sharply declined after 2013 (from 23.7% in 2013 to 6.9% in 2017). There was a smaller reduction in the use of H2RAs after 2013 (from 23.7% in 2013 to 17.9% in 2017). There was a linearly decreasing trend in the use of Gaviscon over the time period (from 20.7% in 2013 to 19.7% in 2017, *β* = −0.44, 95% CI −0.55 to −0.32, *p* < 0.001)). The proportion of infants prescribed with PPI increased more than twofold (4.2% in 2010 to 8.9% in 2017, *β* = 0.60, 95% CI 0.40 to 0.79, *p* < 0.001).

## Discussion

Anti-reflux medications were prescribed to 37% of infants born at <32 weeks’ GA in England and Wales and over 18% were discharged home on these medicines. We found that, on average, anti-reflux medications are started at 32 weeks’ PMA and given for 3 weeks. Our findings show that anti-reflux medication use is more prevalent amongst extremely premature infants. Half of the infants born at <28 weeks’ GA received anti-reflux medication in hospital and a quarter at discharge. This group also received anti-reflux medications for, on average, 9 days more compared to the infants born at 28–31 weeks’ GA.

A wide range of clinical signs/symptoms amongst preterm infants are linked to GORD but none are pathognomonic making it difficult to diagnose clinically. In this database, a diagnosis of GORD was reported in 16% of infants born at <32 weeks’ GA. Previous estimates from UK survey data^10^ report a higher prevalence at 22% in infants born at <34 weeks’ GA. Such surveys, however, report the incidence as estimated by the responding clinician and therefore may not represent true prevalence. We found a lower prevalence reported in the NNRD among <32 weeks’ GA infants while Jadcherla et al.^3^, in a study from 33 hospitals in the United States, reported that 10% of all preterm infants (≤37 weeks GA) had a diagnosis of GORD. The information available in the NNRD does not include how the diagnosis was made and whether any investigations or diagnostic tools were used to determine the presence and severity of GORD. Investigations such as pH monitoring, multichannel intra-oesophageal impedance monitoring, contrast fluoroscopy, endoscopy and biopsy are infrequently used for diagnosis in UK neonatal practice and most cases, are diagnosed based on clinical features or clinical features and a therapeutic trial with anti-reflux medications.^10,11^ Although pH monitoring is available in most neonatal units, less than a third report using it^10^ possibly because it may not be a reliable marker of GORD in preterm infants. The stomach pH of preterm infants is rarely <4 due to frequent milk feeding and a higher baseline pH.^7^ Moreover, higher acidity in the oesophagus does not correlate with symptoms of GORD in newborn infants.^13^

Most infants had GORD diagnosis later in the course of their neonatal stay. The median (IQR) of PMA at first GORD diagnosis was 36 weeks (33–38). Difficulties with feeding such as vomiting, regurgitation or discomfort after large volume feeds often manifest only after the establishment of higher feed volumes. Airways are often protected with devices such as endotracheal tubes and breathing supported with additional respiratory support in the early days after birth. Cardio-respiratory events such as episodes of desaturations may manifest later. These signs may be taken to be suggestive of GOR and may prompt trials of treatment and diagnosis of GORD.

When comparing infants who had a recorded diagnosis of GORD with those who did not, we found that those with GORD diagnosis were more premature and lighter at birth. This is consistent with studies showing that the occurrence of GOR and GORD were higher in younger preterm infants as compared to more matured preterm infants, as well as in lower birth weight infants.^3^ This could be because mechanisms of GORD such as transient lower oesophageal sphincter relaxation (TLESR) are more frequent in more premature infants.^13^ TLESR can lower oesophageal sphincter pressure to levels at, or below, intragastric pressure causing regurgitation of stomach contents. In terms of physiological or structural maturity, less mature infants have lower oesophageal peristaltic velocity as well as a shorter oesophagus and lower oesophageal sphincter (LES) which would exacerbate GOR events more than in more mature infants.^7^ Other factors related to preterm infants’ risk of GORD are relatively abundant milk intake, body position and cow’s milk protein allergy.^13^ Non-pharmacological methods that address some of these can reduce the use of H2RA and PPIs.^14^

Distinguishing physiological GOR and pathological GORD is challenging. Symptoms related to GORD such as excessive crying, regurgitation/vomiting, irritability and back arching frequently occur in healthy infants, and there is also no evidence that these symptoms are temporally associated with GOR events.^15^ Similarly cardio-respiratory events often linked to GORD such as apnoea, episodes of oxygen desaturation and swallowing dysfunction are common in all preterm infants and studies show that suspected clinical reflux behaviours do not correlate with acid reflux as measured by oesophageal multichannel impedance.^15^

Interestingly, we found a higher prevalence of use of anti-reflux medication compared to the prevalence of GORD diagnosis. This suggests that anti-reflux medications are prescribed without the diagnosis of GORD such as for a trial of a therapy which may be used as a diagnostic tool. A survey of UK clinicians showed that 50% of the respondents regularly used clinical features plus therapeutic trials to determine the diagnosis of GORD.^10^ Trials of anti-reflux medications to establish a diagnosis of GORD are recommended in older infants and children^1^ but such therapeutic trials are not recommended in preterm infants because clinical signs and symptoms do not correlate with acidic or non-acidic reflux and the signs improve with time without treatment.^7^ Such empirical treatments can alter the gut microbiome enhancing pathogenic flora and increase the risks of NEC and infections.^6^ Shakeel et al.^14^ reported large reductions in the use of anti-reflux medications with the implementation of guidelines that recommended conservative and expectant management of GOR symptoms until preterm infants were 37 weeks PMA, demonstrating that standardised clinical practice guidelines, with appropriate education that addresses knowledge gaps in GORD management, can reduce inappropriate use of anti-reflux medications.

Although Gaviscon, a preparation of sodium alginate combined with sodium bicarbonate, was the most prescribed among the total cohort (57% of all infants <32 weeks’ GA), H2RAs, mainly ranitidine, use was more prevalent among the extremely preterm infants, prescribed to 64%. In addition, 20% received PPIs. These frequencies of use are higher, participially for H2RA use, than that reported from 43 US hospitals between 2006 and 2013 where, among infants <29 weeks’ GA, 24% had any H2RA and 17% had any PPI.^8^

Not only did a larger proportion of extremely preterm infants receive anti-reflux medications, they also received them for longer. The median duration of anti-reflux prescription was 9 days longer for those born at <28 weeks’ GA as compared to those born at 28–31 weeks’ GA. The US study recorded median (IQR) treatment duration of 15 (6–35) days but this included preterm infants of 26–36 weeks GA.^8^ The longer use of medications among extremely preterm infants could possibly be due to a more prolonged course of GORD due to greater immaturity or because they usually stayed longer in the neonatal unit which opens a longer window of opportunity for medication use as compared to those who were born at later gestations.

It was alarming to note the high use of prokinetics which was prescribed to 45% of the cohort considering that the warning issued by the Food and Drug Administration (FDA) in 2009 and by the European Medicines Agency (EMA) in 2013^1^ which clearly stated that their potential side effects counterweigh the possible benefits of these medications for the treatment of GORD in infants <12 months of age.^16^ It was, therefore, reassuring that the use has decreased significantly, particularly since 2013, perhaps in keeping with the release of the EMA statement and, later, the UK Medicine and Healthcare products Regulatory Authority (MHRA) announcement on the risk of adverse cardiac events with domperidone.^1^

The use of H2RAs and Gaviscon also declined after 2013, although not as rapidly. At this time, emerging evidence suggested that the use of H2RA is associated with higher risks of adverse effects including infections and NEC^17,18^, although this was already mentioned in earlier guidelines.^16^ Gaviscon, perhaps seen as more innocuous due to lack of evidence of such associations also continues to be used frequently. It can decrease the number of acidic GOR episodes, total oesophageal acid exposure and the frequency of regurgitation events^19,20^ but there is no evidence to show that it alleviates clinical signs and symptoms.^19,21^

The use of PPIs has increased more than twofold during the study period. A study conducted by Omari et al.^22^ showed that omeprazole, a PPI, is effective in reducing the frequency of acid reflux episodes and oesophageal acid exposure but has no impact on clinical signs/symptoms in preterm infants. Moore et al.^23^ showed that omeprazole reduced the reflux index as compared to placebo, but changes in clinical features were the same in both groups. These findings are similar to studies using other PPIs such as lansoprazole and esomeprazole.^24,25^

The use of the NNRD as source data has limitations as it is not used for primary prescribing, and diagnoses are not recorded in a standardised manner such as using ICD codes. There is a risk of data entry errors and missing data. The lower prevalence in NNRD data than the use of anti-reflux medications could also reflect diagnoses not recorded or may represent the lack of formal diagnosis. Additionally, due to the limited information included in the database, we are unable to investigate the details of these prescriptions including the dose and frequency of administration, adverse events, response to medications, and why they were stopped which are all vital information required to assess the rational use of any medication. However, NNRD is the only large database of neonatal data in the UK. The robustness and validity of NNRD data have been previously demonstrated and it is increasingly used for research.^26^ The use of this database allowed us to analyse data from over 58,000 infants born at < 32 weeks’ GA representing almost all the eligible population in England and Wales from 2010 to 2017. To our knowledge, this is the first and largest attempt in analysing the prevalence of GORD diagnosis and the prescriptions of anti-reflux medications in England and Wales with data from 200 neonatal units. The results provide valuable information on the use of anti-reflux medications in a large population over a long period of time.

GORD remains an area fraught with diagnostic and management conundrums^27^ and the high prevalence of use of medications despite the lack of evidence of benefit and associations with potential harm, reflects these uncertainties. While large randomised controlled trials are required to evaluate the benefits and harms of using anti-reflux medications in preterm infants, such studies cannot be performed without some clarity or consensus on how to identify infants who have pathological GORD. When perception of “troublesome” symptoms are used to diagnose and treat GORD, trials show no evidence of benefit.^28^ Objective determination of diagnosis such as using the acid reflux index^27^ can identify infants with true oesophageal acid exposure but do not correlate with clinical signs and symptoms. Additionally, these investigations are not used routinely in the UK^10,11^ and therefore findings from studies using such methods of diagnosis may not readily translate to change in practice. Pragmatic studies that investigate methods of identifying infants thought to have “troublesome” GORD, tool to monitor progression of signs and symptoms attributed to GOR, and evaluation of practices that could reduce the use of anti-reflux medications may help achieve rational treatment for preterm infants.

## Conclusion

Our findings suggest that anti-reflux medications are frequently prescribed in very preterm infants despite evidence suggesting that they are not effective and may be harmful. Further research and clear guidelines for diagnosing GORD and for rationalising the pharmacological management of GORD are required.

## Data Availability

Data are available from the corresponding author upon request.
